# Ca^*2*+^ Regulates Dimerization of the BAR Domain Protein PICK1 and Consequent Membrane Curvature

**DOI:** 10.3389/fnmol.2022.893739

**Published:** 2022-06-02

**Authors:** Georgiana F. Stan, Deborah K. Shoemark, Dominic Alibhai, Jonathan G. Hanley

**Affiliations:** ^1^School of Biochemistry, University of Bristol, Bristol, United Kingdom; ^2^Wolfson Bioimaging Facility, University of Bristol, Bristol, United Kingdom

**Keywords:** BAR domain, endocytosis, PDZ domain, calcium, synaptic plasticity

## Abstract

Bin-Amphiphysin-Rvs (BAR) domain proteins are critical regulators of membrane geometry. They induce and stabilize membrane curvature for processes, such as clathrin-coated pit formation and endosomal membrane tubulation. BAR domains form their characteristic crescent-shaped structure in the dimeric form, indicating that the formation of the dimer is critical to their function of inducing membrane curvature and suggesting that a dynamic monomer–dimer equilibrium regulated by cellular signaling would be a powerful mechanism for controlling BAR domain protein function. However, to the best of our knowledge, cellular mechanisms for regulating BAR domain dimerization remain unexplored. PICK1 is a Ca^2+^-binding BAR domain protein involved in the endocytosis and endosomal recycling of neuronal AMPA receptors and other transmembrane proteins. In this study, we demonstrated that PICK1 dimerization is regulated by a direct effect of Ca^2+^ ions *via* acidic regions in the BAR domain and at the N-terminus. While the cellular membrane tubulating activity of PICK1 is absent under basal conditions, Ca^2+^ influx causes the generation of membrane tubules that originate from the cell surface. Furthermore, in neurons, PICK1 dimerization increases transiently following NMDA receptor stimulation. We believe that this novel mechanism for regulating BAR domain dimerization and function represents a significant conceptual advance in our knowledge about the regulation of cellular membrane curvature.

## Introduction

Numerous fundamental cellular processes require changes in membrane curvature to generate tubular or vesicular structures involved in maintaining or modulating the compartmentalization of membrane-bound organelles, transmembrane proteins, and proteins destined for secretion (Mahapatra et al., 2021). In particular, clathrin-mediated endocytosis requires precise and varied changes in plasma membrane curvature to initiate membrane invagination followed by the generation of the neck of the clathrin-coated pit in the form of a short tubular structure (Haucke and Kozlov, 2018). Similarly, endosomal sorting involves the formation of spatially defined membrane tubules and the subsequent generation of transport vesicles (van Weering and Cullen, 2014). Specific protein cargoes and essential accessory proteins must be recruited to these curved membranes in a highly orchestrated manner in order to precisely modulate protein trafficking (McMahon and Boucrot, 2015; Simunovic et al., 2019).

Bin-Amphiphysin-Rvs (BAR) domain proteins are the predominant cellular regulators of membrane curvature and play key roles in endosomal sorting and vesicle biogenesis during endocytosis. Moreover, they function as scaffolds for the recruitment of critical protein machinery and cargo to curved membranes to promote and regulate trafficking events (Daumke et al., 2014; Nishimura et al., 2018). The BAR domain is characterized by a helical bundle that homodimerises in an antiparallel manner, which in the majority of cases results in a crescent-shaped structure that contains positively charged amino acid residues in its concave face that bind negatively charged phospholipids to induce or sense membrane curvature, often in conjunction with modulation of the actin cytoskeleton (Peter et al., 2004; Mesarec et al., 2016). BAR domains vary in size and curvature across the protein family, and the geometry of the BAR dimer is thought to determine the curvature of the membrane that the protein binds (Salzer et al., 2017). The formation of the dimer is assumed to be critical to BAR domain proteins to generate membrane curvature, suggesting that a dynamic dimer–monomer equilibrium, regulated by cellular signaling factors such as Ca^2+^, is a potentially powerful mechanism for controlling BAR domain protein function. To the best of our knowledge, mechanisms for regulating BAR domain dimerization remain unexplored.

PICK1 is a PDZ and BAR domain containing protein whose best-characterized role is to regulate the trafficking of AMPA-type glutamate receptors (AMPARs) in neurons *via* a PDZ interaction with AMPAR subunit GluA2 (Kim et al., 2001; Terashima et al., 2008). Rapid, synapse-specific regulation of AMPAR endocytosis and endosomal sorting in response to Ca^2+^ signaling is critical to synaptic transmission and plasticity (Henley and Wilkinson, 2013; Diering and Huganir, 2018). In particular, PICK1-mediated AMPAR internalization is a key component of NMDA receptor-dependent Long-Term Depression (LTD), a form of synaptic weakening that is essential for numerous forms of learning and memory, and is also involved in several neurological disorders (Kim et al., 2001; Terashima et al., 2008; Li et al., 2016). PICK1 promotes AMPAR internalization *via* a mechanism that involves BAR domain interactions with membrane phospholipids (Jin et al., 2006), and NMDAR-mediated Ca^2+^ influx causes changes in the PICK1 tertiary structure *via* an N-terminal (NT) acidic region that is essential for binding Ca^2+^ ions to modulate PICK1 binding to GluA2 (Hanley and Henley, 2005; Citri et al., 2010). AMPAR endocytosis also requires direct PICK1 interactions with the core endocytic proteins dynamin and AP2, showing functional similarities to the related BAR domain protein amphiphysin (Fiuza et al., 2017).

In this study, we investigated the monomeric–dimeric state of PICK1, and demonstrate that dimerization is increased in the presence of low micromolar [Ca^2+^]. We identified a previously uncharacterized region of acidic amino acids in the PICK1 BAR domain that binds Ca^2+^ ions and is required for Ca^2+^-dependent dimerization. Moreover, we demonstrate that NMDAR stimulation in neurons increases PICK1 dimerization.

## Materials and Methods

### Cell Culture

HEK293 and COS7 cells were cultured in complete Dulbecco’s Modified Eagle Medium (DMEM; Lonza) supplemented with 10% fetal bovine serum (Sigma), 2 mM glutamine (Sigma), and 0.1 mg/ml penicillin/streptomycin (Sigma) and passaged at 70–80% confluency. The cells were transfected with the Lipofectamine 2000 transfection reagent according to the manufacturer’s instructions.

Rat embryonic neuronal cultures were prepared from E18 Wistar rats using standard procedures. The culture medium was Neurobasal (Gibco) supplemented with B27 and 2 mM glutamine (Gibco). Neurons were transfected with plasmid DNA using Lipofectamine 2000 (Thermo Fisher) at DIV 15–16 for FRET pairs and used for experiments 2 days later. For NMDAR stimulation, neurons were incubated with 0.5 μM TTX for 30 min and then stimulated with 30 μM NMDA (Tocris), 20 μM glycine, and 0.5 μM TTX for 3 min in HEPES-buffered saline (25 mM HEPES pH7.4, 140 mM NaCl, 5 mM KCl, 1.8 mM NaCl, 0.8 mM MgCl_2_, 1.8 mM CaCl_2_, and 10 mM glucose).

### DNA Constructs

His_6_ proteins were expressed from pET28 (Novagen). For HEK293, COS7 cell, and neuronal experiments, mGFP-PICK1 and mCherry-PICK1 were expressed from modified pEGFP-C1 or pcDNA3.1. Amphiphysin2-GFP was a gift from Dr. Emmanuel Boucrot and GFP-endophilin was a gift from Dr. Ira Milosevic. Site-directed mutagenesis was carried out by PCR using a KOD HotStart polymerase kit (Merck) and primers from Sigma.

### Preparation of Purified Proteins

Bacterial expression of his_6_-tagged proteins was carried out using BL21(DE3) (Agilent) bacteria transformed with pET vectors cultured in LB containing kanamycin. Cultures were grown to an optical density (OD_600*nm*_) of 0.7–1 and expression induced for 3–4 h with 1 mM IPTG at 30°C. Bacteria were lysed in 150 mM KCl, 50 mM HEPES pH 7.4, 10% glycerol, 1 mM DTT, 25 mM imidazole, 1% Triton X-100, and EDTA-free protease inhibitors (Roche) aided by sonication. After purifying by centrifugation, the extracts were incubated with Ni-NTA beads (Qiagen) for 3 h at 4°C. After washing, protein was eluted with buffer containing 200 mM imidazole pH 6.8.

### DSS Cross-Linking and Manipulation of [Ca^2+^]

To increase intracellular [Ca^2+^], cells were incubated in 140 mM NaCl, 5 mM KCl, 25 mM HEPES pH 7.4, 10 mM glucose, 1–5 mM CaCl_2_, and 3 μM ionomycin (Cayman Chemicals) for 5 min at 37°C.

Cross-linking of proteins in HEK293 cells was carried out in 2 ml PBS (GE Lifesciences) with 0.3 mM DSS (Thermo) for 20 min at room temperature. After the reaction was quenched with 50 mM Tris for 15 min, the cells were lysed in 150 mM NaCl, 20 mM HEPES, 0.5% Triton X-100, and EDTA-free protease inhibitors (Roche). Lysates were cleared by centrifugation and subsequently analyzed by Western blotting.

For cross-linking of his_6_PICK1, 100 nM purified protein was diluted in 150 mM NaCl, 20 mM HEPES pH 7.4, 5 mM HEDTA, and CaCl_2_ at a concentration calculated using the MaxChelator online tool to give required [Ca^2+^]_*free*_. [Ca^2+^]_*total*_ were 0.75 mM, 1.5 mM, 2.5 mM, and 3.4 mM to give [Ca^2+^]_*free*_ of 2, 5, 12, and 26 μM, respectively. 10 μM DSS was added for 20 min at room temperature before the reaction was quenched with 50 mM Tris for 15 min. The samples were subsequently analyzed by Western blotting.

### Western Blotting

Cell lysates or protein samples were resolved on 7% SDS-PAGE gels and transferred to PVDF using a wet transfer apparatus and blocked in 5% milk solution in PBS-Tween20. The membranes were probed with appropriate primary and secondary antibodies (see below) and the bands were visualized using ECL Western blotting substrates (Thermo Fisher Scientific or GE Healthcare). Where appropriate, the membrane was stripped with Restore Western Blot Stripping Buffer (ThermoFisher) and re-probed. Membranes were incubated with the following primary antibodies: anti-PICK1 (Neuromab) and anti-GFP (Neuromab).

Secondary antibodies conjugated to HRP were obtained from GE Healthcare and used at 1:10,000 dilutions. For densitometry, Western blot films were scanned and analyzed using ImageJ followed by the appropriate statistical analysis carried out using GraphPad Prism. All error bars on graphs represent the standard error of the mean.

### Molecular Modeling

The rat PICK1 sequence was modeled onto the 2.5 Angstrom bar domain crystal structure 1I4D.pdb. The missing loops were modeled using a Chimera-1.14 (Pettersen et al., 2004) loop builder. A single disulfide was formed between adjacent cysteines (for context YS**C**I with LR**C**R) on each monomer once the loops have been built. One calcium ion was placed within reach of the putative calcium-binding site (DDEE) on each monomer.

The resulting sequence for the model was checked using Blast search of the model sequence. Following energy minimization, the PICK1 dimer-calcium complex protein was checked for integrity by Procheck to ensure that no *cis-*peptide bonds or D-amino acids have been formed and 100% residues were observed in the allowed regions of Ramachandran space.

All simulations were performed using GROMACS 2019.4 (Berendsen et al., 1995) and the Amber ff99SB-ILDN (Lindorff-Larsen et al., 2010) forcefield, as NPT ensembles at 310 K under periodic boundary conditions. Hydrogens, consistent with pH7, were added to the complex. Short range electrostatic and van der Waals’ interactions were truncated at 1.4 nm while long range electrostatics were treated with the particle-mesh Ewald’s method and a long-range dispersion correction was applied. A simulation box extending 2 nm from the protein was filled with TIP3P water and 150 mM Na and Cl ions were added to attain a neutral charge overall. Pressure was controlled by using a Berendsen barostat (Humphrey et al., 1996) and temperature was controlled by using a V-rescale thermostat. The simulations were integrated with a leap-frog algorithm over a 2 fs time step, constraining bond vibrations with the P-LINCS method. The structures were saved every 0.1 ns for analysis for 100 ns runs. Simulation data were accumulated on the Bristol BrisSynBio supercomputer Bluegem. Molecular graphics manipulations and visualizations were performed using VMD-1.9.3 (Humphrey et al., 1996) and Chimera-1.14 (Pettersen et al., 2004).

### COS-7 Cell Tubulation Assays

COS-7 cells expressing GFP-tagged proteins were incubated in HBS containing 0, 1, 3, or 5 mM CaCl_2_ and 3 μM ionomycin for 15 min at 37°C to generate graded intracellular [Ca^2+^]. The cells were washed with PBS before fixation with 4% paraformaldehyde. Confocal imaging of 20 randomly selected cells per condition was carried out using the 63 × and the 100 ×, N.A 1.4 oil immersion lenses at 490–550 nm with gating between 0.3 and 8, with an optimized pixel size of 70 nm and a line average of 6 scans. Z-stacks were acquired and maximum intensity projections were generated. The experimenter was blinded to the condition being analyzed, and cells were designated as tubulated if they contained more than two tubular structures detectable by eye. The tubulated cells were analyzed further in order to detect the average number and the length of tubules per cell. We developed a semi-automated script based on a Modular Image Analysis plugin for FIJI (ImageJ). The manual component of the analysis involved selecting areas of suitable saturation within the cytoplasm and the exclusion of the plasma membrane. The automated component applied two filters to the images, namely, a median filter with a radius of 1 px and a “difference of Gaussian” filter with a radius of 2 px, before using the Ridge Detection plugin for the detection and quantification of tubule data. The settings for ridge detection included a threshold of 25 pixels (approximately 1.8 μm for my dataset) for minimum tubule length, intensity thresholds between 6 and 10, and sigma 1.8. Data were normalized and statistical analysis was carried out using GraphPad Prism. To label membrane tubules originating from the cell surface, FM4-64FX (Thermo) was added to cells together with ionomycin. All other aspects of the protocol were the same as above. Confocal image acquisition was at 490–550 nm for visualizing *^GFP^*PICK1 and at 650–750 nm for FM4-64FX.

### FLIM–FRET Data Acquisition and Analysis

Fluorescence lifetime images were acquired using a Leica SP8 CLSM system attached to a DMi8 inverted microscope (*Leica* Microsystems). Excitation was performed by the 488 nm line from a pulsed white light laser with a repetition rate of 80 MHz. Images were acquired using a 63x/1.4NA oil objective, with fluorescence collected within the 490–550 nm window (cells were inspected for mCherry expression at 600–650 nm prior to FLIM), and a notch filter centered at 488 nm minimized any laser scatter into the detector. Time-resolved data were acquired by using a PicoHarp 300 TCSPC module (*PicoQuant*) controlled through the SymPhoTime software (*PicoQuant*). FLIM images were acquired with 512 × 512 pixels and 4,096 time bins. Total integration time per image was approximately 2 min. Data analysis was carried out using the FLIMfit 5.1.1 fitting software tool developed at Imperial College London with the pixel-wise fitting algorithm (Warren et al., 2013). The data were spatially binned to 2 × 2 and temporally binned to 32 ps/bin to ensure sufficient photons per pixel for fitting. The data were cropped between 500 and 12,000 ps to exclude scattered marginal data and the threshold was set at an integrated minimum of 100 counts/pixel. Data were fitted with a single exponential model pixel wise for all pixels above the set threshold. The pixel-wise data were used to generate lifetime heatmaps showing the degree and location of the decline in measured lifetime in an intensity-weighted manner and the data were exported as an average lifetime per image. Data were analyzed for statistical significance using GraphPad Prism.

### Statistical Analysis

**FIGURE 1:**
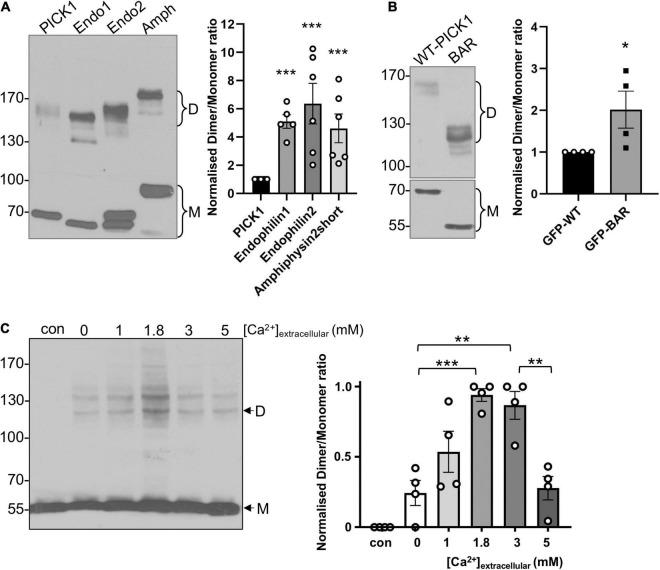
PICK1 BAR domain dimerization is regulated by intramolecular interactions and by Ca^2+^ in heterologous cells. **(A)** A smaller proportion of PICK1 is dimeric compared to endophilin and amphiphysin. HEK293 cells expressing *^GFP^*PICK1, *^GFP^*Endophilin A1 or A2, or *^GFP^*Amphiphysin were treated with 0.3 mM DSS. After quenching of DSS with Tris, the cells were lysed and proteins were detected by Western blotting using anti-GFP. Relative dimer to monomer band intensities were calculated. *N* = 6; ***p < 0.001. **(B)** The isolated BAR domain dimerizes more efficiently than full-length PICK1. HEK293 cells expressing *^GFP^*WT-PICK1 or ^ GFP^PICK1 BAR domain were treated with 0.3 mM DSS. After quenching of DSS with Tris, the cells were lysed and proteins were detected by Western blotting using anti-GFP. Relative dimer to monomer band intensities were calculated. *N* = 4; *p < 0.05. **(C)** HEK293 cells overexpressing untagged PICK1 were incubated in a range of extracellular [Ca^2+^] plus ionomycin for 5 min, followed by 0.3 mM DSS for 20 min. The cells were lysed and proteins were detected by Western blotting using anti-PICK1. Relative dimer to monomer band intensity ratios were calculated. *N* = 4; **p < 0.01, ***p < 0.001; “con” is without DSS.

**FIGURE 2:**
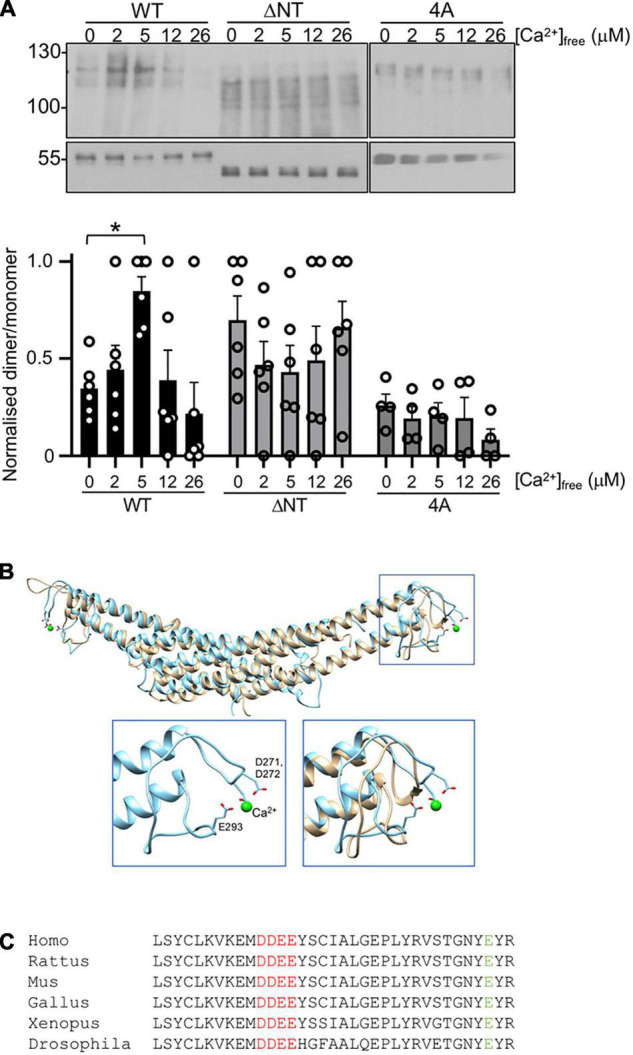
Ca^2+^-dependent PICK1 dimerization is mediated by novel and established Ca^2+^-binding sites. **(A)** 100 nM his_6_WT-PICK1, his_6_ΔNT-PICK1, or his_6_4A-PICK1 were incubated in a range of [Ca^2+^]_*free*_ (buffered with HEDTA) with 10 μM DSS for 20 min. Protein was detected by Western blotting using anti-PICK1. Lower panels show monomers. Relative dimer to monomer band intensities were calculated. The graph shows dimer/monomer ratios, normalized to the highest value in each experiment N = 6, *p < 0.05. **(B)** Molecular modeling predicted that ^271^DD^272^ binds Ca^2+^. Final frames from 100 ns atomistic dynamic simulations demonstrate that ^271^DD^272^, in conjunction with E^293^, binds a Ca^2+^ ion (green sphere). The overlay of WT and 4A shows an intact BAR domain structure in 4A. Insets show the zoom of boxed section, WT-PICK1 alone (left), and WT overlayed with 4A-PICK1 (right). **(C)** Sequence alignment of PICK1 from Homo sapiens, Rattus norvegicus, Mus musculus, Xenopus laevis, and Drosophila melanogaster.

Statistical analysis was carried out using GraphPad Prism version 7.0. The number of individual repeats is indicated in the figure legends, and also on the graph itself with a single dot representing an experimental repeat. Data were presented as mean values ± standard error of the mean (SEM). Western blot data were normalized after calculating dimer–monomer ratios. For comparisons between different proteins (Figures 1A,B), values were normalized to GFP-PICK1; for Ca^2+^-dependent experiments (Figures 1C, 2A), values were normalized to the highest value in each experiment. Multiple *t*-tests were carried out to analyze the differences between PICK1 and other BAR domain proteins, with Bonferroni’s correction for repeated tests. Two-way ANOVAs were carried out to analyze the differences between Ca^2+^ conditions. One-way ANOVAs were carried out to analyze the differences between conditions in the FLIM–FRET experiments followed by Tukey’s *post hoc* analysis.

## Results

### PICK1 BAR Domain Dimerization Is Regulated by Intramolecular Interactions and by Ca^2+^ in Heterologous Cells

To investigate PICK1 dimerization in a cellular context, we used the membrane-permeant cross-linking agent disuccinimidyl suberate (DSS) to cross-link protein complexes in HEK293 cells, and hence to analyze relative levels of monomeric and dimeric PICK1. Using this approach, we compared the basal dimerization state of PICK1 with that of the related BAR domain proteins endophilin and amphiphysin. Cells expressing *^GFP^*PICK1, *^GFP^*endophilin, or *^GFP^*amphiphysin were incubated with 0.3 mM DSS for 20 min, and complexes were subsequently analyzed by Western blotting. While the most abundant species under these conditions was monomeric *^GFP^*PICK1 (∼70 kDa), a clear band was also seen at ∼140–150 kDa, corresponding to dimeric *^GFP^*PICK1. The dimer was the predominant multimer observed using this system; we observed barely detectable levels of tetramer or higher order complexes. Interestingly, a significantly smaller proportion of *^GFP^*PICK1 was in a dimeric state compared to either endophilin or amphiphysin (Figure 1A). Importantly, similar numbers of primary amine groups, which react with DSS, are present in the different BAR domains. This provided initial support for our hypothesis that PICK1 dimerization may be subject to dynamic regulation, i.e., the dimerization is relatively low under basal conditions, and can be upregulated in response to intracellular signaling. Since BAR domain proteins dimerize *via* the BAR domain, we also analyzed the dimerization state of the isolated PICK1 BAR domain in the same system. *^GFP^*BAR domain showed significantly higher levels of dimerization compared to full-length *^GFP^*PICK1 (Figure 1B), suggesting that intramolecular protein interactions inhibit dimerization in the full-length protein.

PICK1 is a Ca^2+^-binding protein, and it has been demonstrated that [Ca^2+^] affects the 3D structure of the protein (Citri et al., 2010). Therefore, we hypothesized that PICK1 dimerization is regulated by Ca^2+^. To test this, we used the Ca^2+^ ionophore ionomycin in conjunction with a range of extracellular [Ca^2+^] ([Ca^2+^]_*extracellular*_) to generate graded Ca^2+^ influx in HEK293 cells, followed by DSS cross-linking to analyze the dimerization of untagged PICK1. Monomeric untagged PICK1 runs at ∼55 kDa, with the corresponding dimer at ∼120 kDa (Figure 1C). This experiment demonstrated that PICK1 dimerization is Ca^2+^-dependent, with relatively low levels of dimerization in the absence of Ca^2+^ influx and maximal dimerization seen at 3 mM [Ca^2+^]_*extracellular*_ (Figure 1C). Furthermore, dimerization shows a biphasic Ca^2+^-sensitivity, with dimerization at 5 mM [Ca^2+^]_*extracellular*_ indistinguishable from basal levels. Additional bands were observed in some experiments, presumably reflecting PICK1 in complex with other HEK293 cell proteins.

### Dimerization of Purified his_6_PICK1 Is Regulated by Ca^2+^ Binding to Two Distinct Sites

A Ca^2+^-dependent increase in dimerization observed in cells could be a direct effect of Ca^2+^ on the PICK1 protein, or alternatively Ca^2+^-dependent intracellular signaling pathways could be involved. We tested the hypothesis that Ca^2+^ has a direct effect on dimerization by performing DSS cross-linking of purified his_6_PICK1 in solutions containing buffered [Ca^2+^]_*free*_ in the low micromolar range. In agreement with the HEK293 cell data, dimerization of his_6_PICK1 showed a biphasic Ca^2+^ dependence, with the maximal dimerization seen at 5 μM [Ca^2+^]_*free*_ and reduced dimerization above 12 μM [Ca^2+^]_*free*_ (Figure 2A). This indicates that PICK1 dimerization is regulated by a direct effect of Ca^2+^ ions on the protein.

**FIGURE 3:**
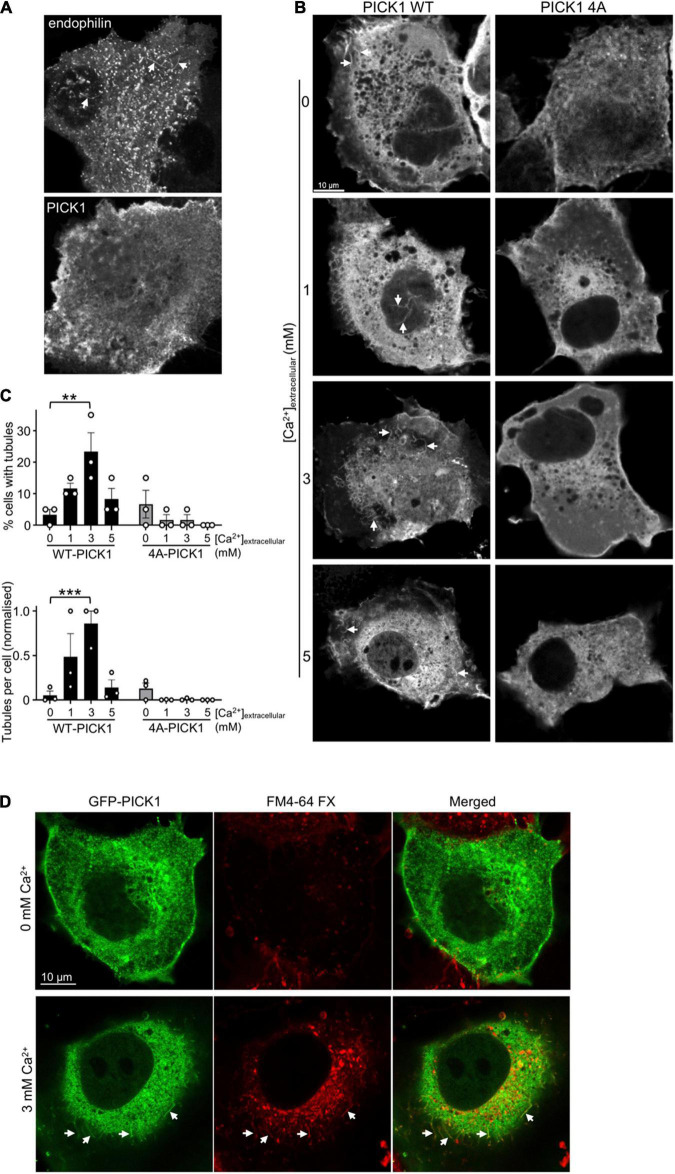
Ca^2+^ stimulates cellular membrane tubulation *via* PICK1 dimerization. **(A)** COS7 cells expressing *^GFP^*PICK1 or *^GFP^*endophilin were fixed and analyzed by confocal microscopy. GFP-positive tubular structures are detectable in cells expressing GFP-endophilin (arrowheads), but not in cells expressing *^GFP^*PICK1. **(B)** COS7 cells expressing *^GFP^*WT-PICK1 or *^GFP^*4A-PICK1 were incubated in 0, 1, 3, or 5 mM extracellular Ca^2+^ and 3 μM ionomycin for 15 min prior to fixation and confocal microscopy. Arrows show tubular structures. **(C)** Quantification of the experiment is shown in B. Tubulation was analyzed as described in the “Materials and methods” section; 20 cells were analyzed per condition in each of three independent experiments. The top graph shows the percentage of cells with detectable tubules, and the bottom graph shows the mean number of tubules per cell. *N* = 3, **p < 0.01, ***p < 0.001. **(D)** *^GFP^*PICK1 tubules originate from the plasma membrane. Cells were treated as in B, with the lipophilic dye FM4-64FX added at the same time as ionomycin. Confocal microscopy demonstrates the colocalization of *^GFP^*PICK1 with FM4-64FX. Arrows show tubular structures.

To investigate the region of PICK1 that mediates Ca^2+^-dependent dimerization, we considered the previously reported N-terminal Ca^2+^-binding site D^4^LDYDIEED^12^, which has been shown to mediate Ca^2+^-dependent binding to the AMPAR subunit GluA2 during LTD (Hanley and Henley, 2005; Citri et al., 2010). Deletion of this acidic domain in his_6_PICK1 [ΔNT-PICK1 (Hanley and Henley, 2005)] caused an increase in basal dimerization (zero Ca^2+^), and occluded further Ca^2+^-dependent increase in dimerization (Figure 2A). In addition, we considered a region of acidic amino acids ^271^DDEE^274^ in the BAR domain, located between helices 2 and 3 at the outer extremity of the dimeric complex (Figures 2B,C). Since this site has not been studied previously, we investigated its theoretical Ca^2+^ binding using molecular modeling; 100 ns atomistic dynamics simulations indicate that a Ca^2+^ ion remains bound to ^271^DD^272^ in conjunction with E^293^ throughout the simulation, as shown by the last frame inset in Figure 3B. Under comparable simulation conditions (starting with Ca^2+^ ions in the same positions) when residues ^271^DDEE^274^ are substituted by alanines (4A-PICK1), the Ca^2+^ ions do not remain bound. The overall fold of the PICK1 BAR domain is not disrupted by this mutation (Figure 2B). ^271^DDEE^274^ and E^293^ are highly conserved in vertebrates and flies (Figure 2C).

In DSS cross-linking assays, while his_6_4A-PICK1 showed a similar level of basal dimerization (zero Ca^2+^) compared to WT-PICK1, dimerization of 4A-PICK1 was unaffected by increasing [Ca^2+^] (Figure 2A). These experiments demonstrate that the Ca^2+^-dependent increase in PICK1 dimerization requires direct binding of Ca^2+^ ions to the ^271^DDEE^274^ acidic region in the BAR domain.

### Ca^2+^-Dependent PICK1 Dimerization Induces Cellular Membrane Tubulation

The primary role of BAR domain proteins is to generate membrane curvature (Daumke et al., 2014; Nishimura et al., 2018), and previous reports have demonstrated that various BAR domain proteins overexpressed in COS7 cells cause the formation of membrane tubules that originate from the plasma membrane (Peter et al., 2004; Chang-Ileto et al., 2011; Myers et al., 2016). To the best of our knowledge, induction of membrane curvature by PICK1 has not been demonstrated previously. To investigate whether PICK1 can generate membrane curvature, we expressed *^GFP^*PICK1 or *^GFP^*endophilin in COS7 cells. While cells expressing *^GFP^*endophilin contained GFP-positive tubular structures, consistent with previous studies (Chang-Ileto et al., 2011; Myers et al., 2016), the *^GFP^*PICK1 expressing cells rarely showed detectable tubules (Figure 3A). To test the hypothesis that the generation of membrane curvature by PICK1 requires an increase in [Ca^2+^], we used ionomycin to increase intracellular [Ca^2+^] as above. In the presence of ionomycin and 3 mM [Ca^2+^]_*extracellular*_, the proportion of *^GFP^*WT-PICK1 expressing cells with tubules increased significantly (Figures 3B,C). Furthermore, the mean number of tubules per cell was also significantly higher at 3 mM [Ca^2+^]_*extracellular*_ compared to zero Ca^2+^ (Figure 3C). In contrast, cells expressing *^GFP^*4A-PICK1, whose dimerization is unaffected by [Ca^2+^], showed undetectable levels of tubulation (Figures 3B,C). To investigate whether the observed structures were membrane tubules that originated from the cell surface, we applied the fixable lipophilic dye FM4-64FX to cells during ionomycin treatment. Colocalization of *^GFP^*PICK1 with the FM4-64FX fluorescence signal demonstrated that *^GFP^*PICK1 induced the formation of membrane tubules from the plasma membrane in the presence of Ca^2+^ (Figure 3C).

### NMDA Receptor Stimulation Increases PICK1 Dimerization in Neuronal Dendrites

**FIGURE 4:**
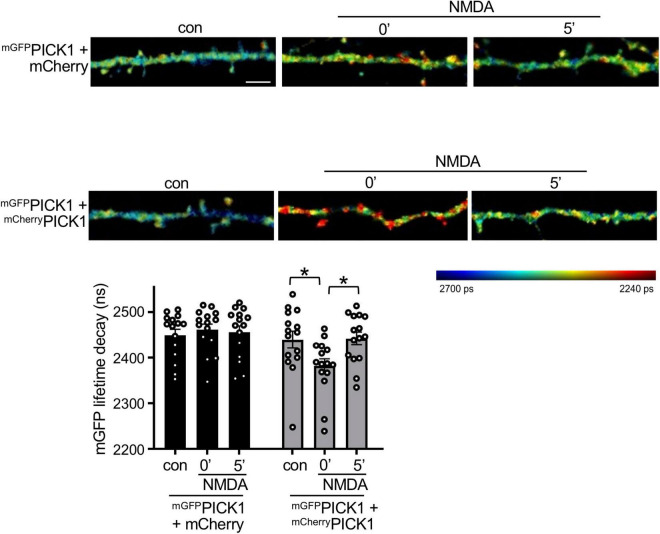
NMDAR stimulation increases PICK1 dimerization in neurons. DIV17-18 neurons expressing *^mGFP^*PICK1 alone, *^mGFP^*PICK1 and free mCherry, or *^mGFP^*PICK1 and *^mCherry^*PICK1 were stimulated with 30 μM NMDA or vehicle for 3 min, followed by fixation immediately after stimulation or 5 min after removal of NMDA. The lifetime decay of GFP was fitted (see the “Materials and methods” section) and the representative intensity merged, fluorescence lifetime images of neuronal dendrites are shown. Red color corresponds to short lifetime, therefore high FRET and increased PICK1 dimerization. The colored bar on the right-hand side represents the lifetime of mGFP ranging from 2,240 ps (red) to 2,700 ps (blue). The lifetime signal is intensity-adjusted and the scale bar is consistent between panels at 5 μm. The graph shows mean recorded lifetimes; *n* = 15 cells from five independent experiments, *p < 0.05, one-way ANOVA.

Since PICK1 is required for NMDAR-dependent plasticity in neurons, and our cross-linking data indicate that PICK1 dimerization is Ca^2+^-dependent, we hypothesized that PICK1 dimerization increases in response to NMDAR stimulation. To investigate PICK1 dimerization in neurons, we expressed *^mGFP^*PICK1 and *^mCherry^*PICK1 in dissociated neurons in culture and used FLIM–FRET (Fluorescence Lifetime Imaging–Forster Resonance Energy Transfer) to analyze the association of the two recombinant monomers. As a control, and to provide a baseline for non-interacting proteins, we co-expressed *^mGFP^*PICK1 with free mCherry. Importantly, control neurons expressing *^mGFP^*PICK1 and mCherry showed no change in the FRET signal after NMDAR stimulation (Figure 4). Unstimulated neurons expressing *^mGFP^*PICK1 and *^mCherry^*PICK1 showed a trend toward a shorter mGFP fluorescence lifetime compared to controls, which did not reach statistical significance, suggesting weak FRET between mGFP and mCherry in the PICK1 dimer under basal conditions. However, stimulation with 50 μM NMDA for 3 min caused a significant decrease in mGFP fluorescence lifetime in neurons expressing *^mGFP^*PICK1 and *^mCherry^*PICK1 immediately after stimulation (0 min), indicating an increase in FRET, and therefore an increase in dimerization between the two recombinant proteins (Figure 4). This effect was transient, with a return to basal FRET levels by 5 min after the end of the NMDA stimulation period.

## Discussion

In this study, we demonstrate that the dimerization state of the BAR domain protein PICK1 is regulated by Ca^2+^ ions. We present the unexpected finding that PICK1 is not a constitutive dimer, but instead it can exist in monomeric form in cells, and dimerization is enhanced by increases in [Ca^2+^]. Ca^2+^-dependent dimerization can be observed *in vitro* with purified proteins, indicating a direct effect of Ca^2+^ ions on the PICK1 protein, and also in response to NMDAR stimulation in neurons, suggesting a role in NMDAR-dependent plasticity.

To the best of our knowledge, there have been no previous studies reporting the regulation of BAR domain protein dimerization directly. Nevertheless, a previously published report supports our observation that PICK1 is not an obligate homodimer. It has been demonstrated that PICK1 forms heterodimers with the BAR domain protein ICA69 in overall preference to PICK1 homodimers (Cao et al., 2007). However, the subcellular distribution of ICA69 did not match that of PICK1 when analyzed by immunocytochemistry (Cao et al., 2007), suggesting that PICK1 homodimerization is location-dependent and may be dynamically regulated. Consistent with this report, our FLIM–FRET results suggest that PICK1 homodimerization is localized to clusters in dendrites that may be dendritic spines. This observation suggests a role for NMDAR-dependent increase in PICK1 dimerization close to synapses to promote AMPAR internalization. Furthermore, the transient nature of the observed NMDAR-dependent dimerization suggests a requirement for PICK1 in curvature generation during the first 5 min after stimulation. A further study is required to determine the physiological role of Ca^2+^-dependent PICK1 dimerization in synaptic plasticity.

Cellular membrane tubulation is widely used as an assay for BAR domain-dependent generation of membrane curvature (Peter et al., 2004; Chang-Ileto et al., 2011; Myers et al., 2016), yet such activity has not been reported previously for PICK1. We propose that the absence of such findings to date is due to the low dimerization level of PICK1 in cells under basal Ca^2+^ conditions, which is insufficient to generate membrane curvature. It is known that PICK1 is required for NMDA-dependent AMPAR endocytosis (Fiuza et al., 2017), and we showed here that the PICK1-dependent membrane tubules originate from the cell surface in the presence of Ca^2+^. This suggests that Ca^2+^-dependent PICK1 dimerization may be a critical regulatory step in AMPAR endocytosis. PICK1 has been implicated in endosomal sorting events and endocytosis (Lin and Huganir, 2007), and our data do not rule out the possibility that Ca^2+^-dependent PICK1 dimerization is involved in regulating the trafficking of cargo proteins through the endosomal system, perhaps by contributing to the regulation of endosomal tubule biogenesis and consequent recycling or lysosomal targeting.

It was surprising that we did not detect higher order PICK1 oligomers in our experiments in cells or in the reduced system. A previous study demonstrated the existence of presumed tetrameric, hexameric, and higher oligomers following cross-linking with 1 mM DSS in COS7 cells (Karlsen et al., 2015). A possible explanation for this discrepancy is that Karlsen et al. (2015) used 1 mM DSS, whereas we used 0.3 mM. In addition, we carried out DSS cross-linking assays in HEK293 cells; perhaps some unknown aspect of COS7 cell biology favors the formation of higher order oligomers.

PICK1 is a known Ca^2+^-binding protein, with two previously identified Ca^2+^-binding sites (Hanley and Henley, 2005; Citri et al., 2010) in addition to ^271^DDEE^274^, which is described for the first time here. Our results suggest a complex mechanism for Ca^2+^-dependent dimerization involving both ^271^DDEE^274^ in the BAR domain and the N-terminal site (D^4^LDYDIEED^12^), whereby D^4^LDYDIEED^12^ inhibits dimerization at basal [Ca^2+^], and this inhibition is relieved by deleting the region or by the presence of increased [Ca^2+^]. In addition, Ca^2+^ binding to ^271^DDEE^274^ promotes dimerization, perhaps by disrupting an intramolecular interaction. It is interesting to note that neither of the regulatory Ca^2+^-binding sites are situated in the dimer interface, suggesting allosteric effects of Ca^2+^ on the PICK1 structure to influence dimerization. Indeed, it has been shown previously that Ca^2+^ binding to D^4^LDYDIEED^12^ changes the structure of PICK1 when analyzed by circular dichroism (Citri et al., 2010). This proposed mechanism is consistent with our observation that the isolated PICK1 BAR domain dimerizes more efficiently compared to the full-length protein, indicating that dimerization is inhibited by flanking regions of PICK1, which would include D^4^LDYDIEED^12^. Furthermore, PICK1 is the only known BAR domain protein with a PDZ domain and its corresponding N-terminal acidic region. If D^4^LDYDIEED^12^ inhibits dimerization, as suggested above, this could explain the low basal dimerization state relative to other BAR domain proteins as shown in Figure 1. The biphasic response to Ca^2+^, with reduced dimerization above 12 μM [Ca^2+^], suggests the involvement of an additional Ca^2+^-binding site with a lower affinity for Ca^2+^ ions, which inhibits dimerization. The PICK1-GluA2 interaction (Hanley and Henley, 2005) follows a similar pattern of [Ca^2+^] dependency to PICK1 dimerization shown here, suggesting that [Ca^2+^] promotes the recruitment of AMPARs to curved membranes *via* PICK1 during endocytosis.

Our findings increase the possibility that other BAR domain proteins could also be regulated by cellular signaling factors. Indeed, some BAR domain proteins in addition to PICK1 have short acidic clusters between helices 2 and 3 in their BAR domains, which might function as Ca^2+^ sensors in a similar manner as we have demonstrated for PICK1. Furthermore, other BAR domain proteins have also been shown to heterodimerize, suggesting the existence of mechanisms to regulate an equilibrium between homo- and heterodimerization (Cao et al., 2007; Wu et al., 2009). A further study is needed to determine whether other BAR domain proteins are regulated by Ca^2+^ or other signaling events, such as phosphorylation.

## Data Availability Statement

The original contributions presented in the study are included in the article/supplementary material, further inquiries can be directed to the corresponding author.

## Ethics Statement

The animal study was reviewed and approved by Bristol University Animal Welfare and Ethical Review Body (AWERB). This work was carried out under the project UIN UB/19/005.

## Author Contributions

GS designed and performed molecular cell biology experiments. DS designed and performed molecular modeling. DA designed and supervised FLIM experiments. JH conceived the project, supervised the project, designed experiments and wrote the manuscript. All authors contributed to the article and approved the submitted version.

## Conflict of Interest

The authors declare that the research was conducted in the absence of any commercial or financial relationships that could be construed as a potential conflict of interest.

## Publisher’s Note

All claims expressed in this article are solely those of the authors and do not necessarily represent those of their affiliated organizations, or those of the publisher, the editors and the reviewers. Any product that may be evaluated in this article, or claim that may be made by its manufacturer, is not guaranteed or endorsed by the publisher.
